# A Potential Role for c-MYC in the Regulation of Meibocyte Cell Stress

**DOI:** 10.3390/cells14100709

**Published:** 2025-05-14

**Authors:** Isabella Boyack, Autumn Berlied, Cornelia Peterson

**Affiliations:** Department of Comparative Pathobiology, Tufts University, North Grafton, MA 01536, USA

**Keywords:** Meibomian gland epithelial cells, sebaceous carcinoma, MYC, integrated stress response, CHOP, apoptosis, fatty acid synthase, lipogenesis

## Abstract

The integrated stress response (ISR) is a key regulator of cell survival, promoting apoptosis through the effector protein CHOP in instances of prolonged or severe stress. The ISR’s role in the initiation and progression of epithelial malignancies has been investigated; however, the ISR has not been evaluated in ocular adnexal sebaceous carcinoma (SebCA). Though uncommon, mortality rates of up to 40% have been reported, and the mechanisms underlying SebCA tumorigenesis remain unresolved; however, *c-MYC* upregulation has been documented. Our objective was to determine the role of MYC in modulating the ISR in the Meibomian gland. Human Meibomian gland epithelial cells (HMGECs) were subject to both pharmacologic and genetic manipulations of MYC expression. Cytotoxicity, proliferation, and changes in protein and gene expression were assessed. Conditionally *MYC*-overexpressing mice were subject to topical 4-hydroxytamoxifen (4-OHT) induction of the eyelids prior to tissue harvest for histology, immunohistochemistry, immunoblotting, and qPCR. MYC-inhibited HMGECs exhibited dose-dependent decreased proliferation, increased CHOP expression, and increased apoptosis. Conversely, *MYC*-overexpressing HMGECs and Meibomian glands from 4-OHT-induced mice demonstrated suppressed CHOP expression, reduced apoptosis, and upregulated fatty acid synthase expression. These results suggest that MYC inhibition induces the ISR and promotes apoptosis, while MYC induction suppresses CHOP expression. High MYC expression may, therefore, serve as a mechanism for SebCA to elude cell death by promoting lipogenesis.

## 1. Introduction

The Meibomian gland is a specialized sebaceous gland in the eyelid that is responsible for producing and secreting meibum, which forms the lipid layer of the tear film. Sebaceous carcinoma (SebCA) in the periocular region, or ocular adnexal sebaceous carcinomas, is an uncommon but aggressive malignancy most commonly arising from the Meibomian gland and accounts for 1–5.5% of all eyelid neoplasms [1]. These tumors infiltrate in a pagetoid pattern and can spread through the epidermis of the eyelid or the conjunctival epithelium. This characteristic can confound diagnosis and lead to mortality rates up to 40% [2,3]. While there is limited knowledge regarding the cellular mechanisms behind the progression of sebaceous neoplasms, overexpression of the oncogene *c-MYC* has been demonstrated in these tumors in both human and canine patients using next-generation sequencing, immunohistochemistry, immunolabeling, quantitative PCR (qPCR), and in situ hybridization approaches [3,4,5]. Further, three high-MYC-expressing primary human ocular adnexal SebCA cell lines exhibited robust proliferative and clonogenic potential while retaining adipophilin expression, a surrogate for sebaceous differentiation [5].

*MYC* is a widely studied protooncogene that is commonly dysregulated in tumor cells [6]. *MYC* is involved in the regulation of diverse cellular processes including proliferation, differentiation, and apoptosis, and in some neoplasms, overexpression of *MYC* has resulted in modulation of the integrated stress response (ISR) [6,7,8,9]. The ISR is a complex cellular pathway that has a major role in maintaining cell survival during stress stimuli [10]. Stressors that are well-characterized activators of the ISR include hypoxia, nutrient deprivation, viral infection, the unfolded protein response (UPR), and oxidative and endoplasmic reticular (ER) stress [6,10,11,12,13,14]. The main regulator protein of the ISR is the alpha subunit of eukaryotic translation initiation factor 2 (eIF2α) [10,15]. Following phosphorylation by one of four main kinases including PKR-like ER kinase (PERK), general control non-derepressible 2 (GCN2), double-stranded RNA-dependent protein kinase (PKR), and heme-regulated eIF2α kinase (HRI), eIF2α is activated [16]. Each kinase responds to different cell stress stimuli [16]. Phosphorylated eIF2α globally represses both cap-dependent and cap-independent translation initiation but allows for specific stress-related mRNAs to be translated, which are crucial for cell survival, such as activating transcription factor 4 (ATF4) [10,17]. If the cell is not able to recover from the stress, then the ISR instead promotes apoptosis, mainly through the activation of the C/ECP homologous protein (CHOP) [18]. Numerous small molecules have been shown to modulate components of the ISR, with tunicamycin representing a potent ISR inducer with a well-documented capacity to upregulate the CHOP protein, and ISRIB (ISR inhibitor) serving as the primary inhibitor of the ISR [19,20].

Activation of the ISR can promote tumorigenesis by facilitating migration and immune escape [21,22,23]. In a mouse model of human epidermal growth factor receptor 2 positive (HER2+) breast cancer, activation of the ISR has been shown to have anti-tumor effects [24]. The phosphorylation of eIF2α by PKR reduced proliferation and activated apoptotic pathways, resulting in reducing tumor growth [24]. However, overexpression of HER2 in human breast cancer, conversely, results in ISR activation and upregulated ATF4 expression with increased cell migration [25]. Overexpression of ATF4 in esophageal squamous cell carcinoma promotes metastasis and has been associated with a poorer prognosis [26]. The ISR also plays a role in endothelial cell survival and angiogenesis [27]. The PERK/ATF4 pathway is involved in the regulation of vascular endothelial growth factor expression as well as fibroblast growth factor-2 and interleukin-6 expression in glucose-deprived human tumor cells, resulting in facilitated angiogenesis [27]. The translation of oncogenic mRNAs such as *SOX2* is increased in skin squamous cell carcinoma due to eIF2α phosphorylation [23]. The ISR plays a complex role in many neoplasms and can have many different outcomes depending on the tissue and cancer phenotype.

Our study aimed to determine whether MYC modulates the ISR in the Meibomian gland and to characterize a potential role for MYC-induced ISR regulation in SebCA tumorigenesis by utilizing both genetic and pharmacologic modulating approaches in normal human Meibomian gland epithelial cells (HMGCEs) and a conditionally *MYC*-overexpressing transgenic mouse model.

## 2. Materials and Methods

### 2.1. In Vitro Small Molecule Modulation of the ISR and Fatty Acid Synthesis

Early passage (P4–16) HMGECs (ATCC, Manassas, VI, USA) were maintained in Keratinocyte Serum-Free Medium (KSFM; Gibco, Waltham, MA, USA) supplemented with BPE (50 µg/mL), human recombinant EGF (5 ng/mL), and normocin (100 µg/mL; InvivoGen, San Diego, CA, USA). All cells were incubated at 37 °C with 5% CO_2_. To assess the ISR in non-neoplastic meibocytes, HMGECs were treated with MYCi361 (Selleck Chemicals, Houston, TX, USA), a small-molecule MYC inhibitor with potent in vitro and in vivo anti-tumor effects, tunicamycin (Cell Signaling Technology, Danvers, MA, USA), or ISRIB (Tocris, Minneapolis, MN, USA) [28]. DMSO (Sigma–Aldrich, St. Louis, MO, USA) served as a vehicle control. In a separate series of experiments, HMGECs were treated with increasing concentrations (0, 0.1, 1.0 10, or 100 µM) of the fatty acid synthase inhibitor C75 (Tocris) for 6 h.

### 2.2. In Vitro Proliferation and Cytotoxicity Assessments

To assess proliferative responses, HMGECs were seeded in 96-well plates at a density of 6.4 × 10^3^ cells per well. An MTT assay was performed on cells treated with a 5× serial dilution (0, 0.05, 0.25, 1.25, 6.25 µM) of MYCi361 for two days, with CyQUANT MTT Assay Kit (ThermoFisher Scientific, Waltham, MA, USA). To assess cytotoxicity, HMGECs were seeded into 12-well plates at a density of 7.4 × 10^4^ cells per well and treated with 2.5 uM Myci361, 12.5 ng/mL tunicamycin, 12.5 nM ISRIB, or DMSO as a vehicle control in supplement and antibiotic-free KSFM. Media were collected at 4, 8, and 24 h, and LDH release was assayed using a colorimetric CyQUANT LDH Cytotoxicity Assay Kit (Invitrogen, Waltham, MA, USA) per the manufacturer’s instructions. Percent cytotoxicity was calculated relative to the provided LDH positive control using the following formula:

% cytotoxicity = ((chemical-treated LDH activity − spontaneous LDH activity)/(maximum LDH activity−spontaneous LDH activity)) × 100, where chemical treatment LDH activity was measured under each experimental treatment condition, spontaneous LDH activity was measured in cells incubated with ultrapure water, and maximum LDH activity was measured in naïve cells.

### 2.3. Immunohistochemistry and Apoptosis Assessments

HMGECs were seeded in 8-well chamber slides at a density of 2 × 10^6^ cells per well. Following an 8 h incubation with MYCi361, ISR activation was evaluated using a primary antibody against phospho-eIF2α (Ser51, catalog # 9721; Cell Signaling Technology) at a dilution of 1:1000 and c-MYC (9E10, catalog # 13-2500; Invitrogen) at a dilution of 1:800 with 3% BSA + 2% normal goat serum in 1X PBS + 0.1% Tween (PBS-T) incubated overnight at 4° C. Slides were then incubated with goat anti-mouse, Alexa Fluor 555 (catalog # A-21424; Invitrogen), and goat anti-rabbit, Alexa Fluor 488 (catalog #A-11008; Invitrogen), at a dilution of 1:1000 with 3% BSA + 2% normal goat serum in PBS-T for 60 min at room temperature (RT). DAPI stain was used to visualize nuclei, and cells were mounted in Prolong Gold Antifade solution (ThermoFisher). For experiments in which HMGECs were incubated with increasing doses of C75, immunolabeling for fatty acid synthase (FASN) at a dilution of 1:1000 (catalog# PA5-22111; Invitrogen) and c-MYC was performed overnight at 4 °C followed by incubation with goat anti-mouse, Alexa Fluor 488 (catalog #A-11001; Invitrogen), and goat anti-rabbit, Alexa Fluor 555 (catalog #A-27039; Invitrogen), at a dilution of 1:1000 for 60 min at RT. DAPI staining and mounting were performed as described above.

Apoptosis in treated HMGECs and in formalin-fixed paraffin-embedded (FFPE) sections of murine eyelid and in human sebaceous carcinomas was evaluated using the Scientific Click-iT TUNEL Assay for In Situ Apoptosis Detection with the Alexa Fluor Kit (ThermoFisher) per the manufacturer’s instructions. TUNEL assays were followed by immunohistochemistry using standard protocols and primary antibodies against CHOP (anti-DDIT3 antibody [9C8], ab11419; Abcam, Waltham, MA, USA) at a dilution of 1:200 overnight at 4 °C followed by incubation with goat anti-mouse, Alexa Fluor 555, at a dilution of 1:1000 for 60 min at RT. DAPI staining and mounting were performed as described above. MYC immunolabeling of FFPE sections of human sebaceous carcinoma specimens was previously performed by the Johns Hopkins Hospitals Department of Pathology clinical laboratory as part of a prior study [4].

### 2.4. MYC Transfection

HMGECs were seeded into a 6-well plate at a density of 2.4 × 10^5^ cells per well until approximately 90% confluent. Cells were transfected with 125, 250, or 500 ng per well of pBabe-c-myc-zeo plasmid (plasmid # 17758, Addgene, Watertown, MA, USA) in antibiotic-free Opti-MEM (catalog# 11514-015, ThermoFisher) using Lipofectamine LTX (catalog # 15338-100, ThermoFisher) and standard protocols [29]. GFP (plasmid # 17446, Addgene) with and without lipofectamine were used as a positive and negative control, respectively. Cells were maintained in Opti-MEM for five days prior to protein or RNA isolation.

### 2.5. Protein Isolation and Western Blotting

Protein isolation for HMGECs was performed with RIPA cell lysis buffer composed of NaCl, Triton X-100, 1 M Tris (pH 8.0), and phosphatase and protease inhibitor cocktails (ThermoFisher). Protein isolation for tissue samples was performed in cell lysis buffer, and tissue was homogenized until there was no gross evidence of tissue remaining. A Pierce BSA assay was performed for protein quantification. Normalized proteins were subject to SDS-PAGE using Bio-Rad Laboratories Inc. (Hercules, CA, USA) Mini-PROTEAN TGX Precast gel (catalog # 456-9033) and were electrically transferred to polyvinylidene difluoride (PVDF) membranes. Membranes were blocked with 5% BSA in tris-buffered saline (TBS) containing 0.01% Tween-20 (TBS-T) for 60 min at RT. Membranes were incubated with primary antibodies diluted 1:1000 in 5% BSA in TBS-T overnight at 4 °C with the following antibodies: GCN2 (catalog #3302; Cell Signaling Technology), PERK (C33E10, catalog #3192; Cell Signaling Technology), CHOP (L63F7, catalog #2895; Cell Signaling Technology), and ß-actin (Invitrogen, catalog #PA1-183-HRP). Membranes were incubated with secondary antibodies (goat anti-mouse HRP, catalog #31437, Invitrogen or donkey anti-rabbit HRP, catalog #31458, Invitrogen) diluted 1:5000 in 5% BSA in TBS-T for 60 min at RT before being visualized with enhanced chemiluminescence (ECL). Densitometry was performed using ImageJ software (v. 1.54k; National Institutes of Health, Bethesda, MD, USA), and protein expression was normalized to ß-actin.

### 2.6. RNA Isolation, cDNA Synthesis, and Quantitative PCR (qPCR)

RNA was extracted from homogenized murine eyelids using a combination TRIzol (ThermoFisher) protocol and an RNeasy Kit (catalog #74104; Qiagen, Germantown, MD, USA), and HMGECs were subject only to the RNAeasy Kit. RNA was stored at −80 °C until further processing. cDNA was synthesized using a SuperScript VILO Kit (ThermoFisher). Reverse transcription and quantitative polymerase chain reaction (qPCR) were conducted using the TaqMan Gene Expression Assays (ThermoFisher) and a QuantStudio 3 System (ThermoFisher). Expression of *MYC*, *CHOP*, and *FASN* was quantified using 2_ΔcT normalized to *polR2a* using predesigned probes: Hs00153408_m1 (human *MYC*), Mm00487804_m1 (murine *MYC*), Hs00358796_g (human *CHOP*), Mm01135937_g1 (murine *CHOP*), Mm00662319_m1 (murine *FASN*), Hs00172187_m1 (human *polR2a*), and Mm01309448_m1 (murine *polR2a*) (ThermoFisher).

### 2.7. Modulation of MYC Expression In Vivo

This study was approved by the Institutional Animal Care and Use Committee (IACUC) of Tufts University. All experiments were performed in accordance with the guidelines for the Use of Animals in Ophthalmic and Vision Research of the Association for Research in Vision and Ophthalmology (ARVO). Information regarding in vivo experiments reported in this manuscript is in adherence with the ARRIVE 2.0 Guidelines [30]. Cryopreserved murine sperm were kindly gifted from Dr. Fiona Watt, and transgenic mice were rederived by the Johns Hopkins University Murine Mutagenesis Core [31]. Animals were maintained in Nexgen 500 individually ventilated cages (Allentown Inc., Allentown, NJ, USA) (*n* ≤ 5 adults/cage) in a temperature and humidity-controlled (70 ± 2.0 °F; 30–70%), 12 h day/night light cycle environment with food (irradiated Teklad Global 18% Rodent Diet; Inotiv, Indianapolis, IN, USA) and water ad libitum. Breeding females and adolescent mice were provided DietGel GEM dietary supplement (Clear H_2_O, Westbrook, ME, USA) to support in the peri-gestational and peri-weaning periods, respectively. Animal care was provided by the Tufts University Division of Animal Resources at the Cummings School of Veterinary Medicine.

Male and female (3 to 13 weeks old) transgenic (TG) C57B6 mice expressing the human *MYC-2* cDNA fused to the hormone-binding domain (ERTM) of a mutant murine estrogen receptor in a keratin 14 (K14) expression cassette (K14MycER) and their wildtype (wt) littermates were used for in vivo evaluations [31]. Animals were screened for the presence of the transgene by PCR of distal tail tissue with the following primers: F: 5′-TACTCTGAGTCCAAACCGGGC-3′; R: 5′-AGCCTGGTAGGAGGCCAGCTTCTCTGA-3′, as previously described [31]. *MYC*-induction was achieved in TG mice (P22–26) through once daily unilateral topical application of 4-hydroxytamoxifen (4-OHT; Sigma–Aldrich) dissolved in ethanol and corn oil to the eyelid skin (10 mg/kg/day) for five days. Corn oil vehicle and 4-OHT-treated wt mice served as non-induced controls. In a subsequent experiment, both wt and TG mice were treated once daily with 4-OHT, as for the initial experiment, followed by either topical application of MYCi361 (50 mg/kg/day) or vehicle control (5% DMSO, 40% PEG300, 5% Tween 80, 50% ddH_2_O) for three days beginning 24 h after the last dose of 4-OHT. Mice were humanely euthanized at P90 regardless of intervention. Eyelids with periocular skin, globes, and dorsal skin were collected and either fixed in 4% PFA for 48 h and then stored in 1X PBS prior to processing for paraffin embedding or freezing for embedding in OCT or were homogenized in RIPA buffer (ThermoFisher) or TRIzol for subsequent protein and RNA extraction.

### 2.8. Histology, Morphometric Analyses, and Immunohistochemistry

Hematoxylin and eosin (H&E)-stained whole mount sections of FFPE and Oil-Red-O-stained sections (StatLab, McKinney, TX, USA) of frozen, OCT-embedded murine eyelid tissue were utilized to visualize the morphology and lipid content of Meibomian glands, respectively. Whole-slide images (WSI) of H&E-stained sections were obtained using a VS200 Research Slide Scanner (Olympus, Westborough, MA, USA) at 40×. The cross sectional area of meibocytes was determined by multiplying the longest dimension measured using the arbitrary line tool in the OlyVIA (Olympus, v.4.2, build 31689) by the orthogonal dimension (Supplementary Figure S1) of ten cells per acinus and four acini per mouse (*n* = 2/upper eyelid; 2/lower eyelid). Only meibocytes with sebaceous differentiation, circumferentially distinct cytoplasmic borders, and nuclei in the plane of section were utilized for obtaining measurements. Routine avidin–biotin complex ABC immunohistochemistry was performed on FFPE murine eyelid using standard techniques. Antigen retrieval was performed using sodium citrate buffer at 95 °C for 10 min. CHOP expression was evaluated using an anti-DDIT3 antibody at a dilution of 1:200 with 3% BSA in PBS-T followed by a biotinylated goat anti-mouse secondary antibody (catalog #31800; ThermoFisher) at a dilution of 1:1000. Antigen was visualized using a 3,3′-diaminobenzidine (DAB) chromogen (ThermoFisher), hematoxylin counterstain, and standard brightfield microscopy using an Olympus BX41 microscope.

### 2.9. Statistical Analyses

Statistical differences in mean MTT expression, percent cytotoxicity, relative protein and transcript expression, and cross-sectional area of murine meibocytes were evaluated using one-way ANOVAs with Dunnett’s post hoc tests for multiple comparisons relative to controls. The IC_50_ for MYCi361 in HMGECs was determined using a nonlinear regression model with the absolute IC50 option using Prism 8 GraphPad (v. 10.4.1; San Diego, CA, USA) (α = 0.05).

## 3. Results

### 3.1. MYC-Inhibited HMGECs Exhibit Diminished Proliferative Capacity, Activation of the ISR, and Increased Cytotoxicity Due to Apoptotic Cell Death

**Figure 1:**
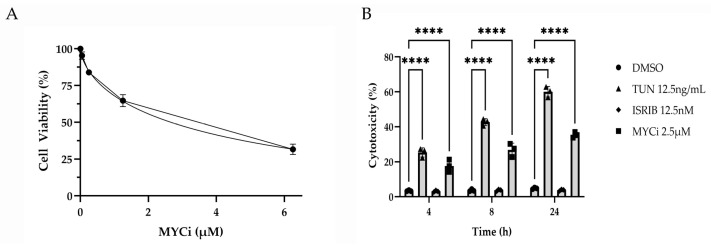
Antiproliferative and cytotoxic effects of MYC inhibition in vitro. An MTT assay performed to assess the dose response of HMGEC proliferation to MYCi361 (MYCi; *n* = 3 wells/concentration) over two days (**A**). Percent cytotoxicity calculated from lactate dehydrogenase (LDH) release measured from HMGEC supernatant following treatment with tunicamycin (TUN), integrated stress response inhibitor (ISRIB), or MYCi (*n* = 3 wells/treatment/time point) relative to the positive LDH control. Percent cytotoxicity of ISR- and MYC-modulated cells was compared to the experimental DMSO control at 4, 8, and 24 h (**B**). **** *p* ≤ 0.0001.

HMGEC proliferation was measured in response to two-day incubation with varying concentrations of Myci361 (0–6.25 µM) using an MTT assay. HMGECs exhibited a dose-dependent decrease in proliferation with an IC_50_ of 2.66 µM (Figure 1A). For subsequent experiments, 2.5 µM MYCi361 was selected to ensure sufficient viable cell mass for downstream analyses. A lactate dehydrogenase (LDH) assay was performed to measure HMGEC cytotoxicity in response to treatment with MYCi, tunicamycin, ISRIB, or DMSO control. LDH is released into the extracellular environment by damaged cells, and quantification of LDH in culture supernatant is commonly utilized as a marker for cytotoxicity [32]. Tunicamycin- (*p* ≤ 0.0001) and MYCi-treated (*p* ≤ 0.0001) cells exhibited significantly greater percent cytotoxicity when compared to the vehicle control. There were no significant differences in LDH release between ISRIB- (*p* = 0.9980) and DMSO-treated cells (Figure 1B).

**Figure 2:**
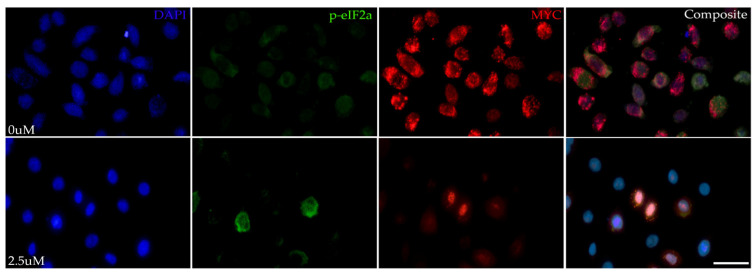
ISR-stimulating effects of MYC inhibition in vitro. Representative photomicrographs of HMGECs incubated for eight hours in DMSO (0 µM) or MYCi361 (2.5 µM) (*n* = 3 chambers/treatment) subject to p-eIF2α and MYC co-immunolabeling. Phospho-eIF2αa expression (Alexa Fluor 488, green) was upregulated in response to MYC suppression (Alexa Fluor 555, red). DAPI (blue). Scale bar: 10 µm.

To determine whether MYC inhibition resulted in ISR modulation, immunolabeling for phospho-eIF2α (p-eIF2α) and MYC was performed on HMGECs following an eight-hour incubation with Myci361 or the vehicle control (DMSO). Suppressed MYC expression was confirmed, and cytoplasmic p-eIF2α expression was upregulated in MYC-inhibited cells relative to the vehicle control (Figure 2), indicating that MYC inhibition activates the ISR.

**Figure 3:**
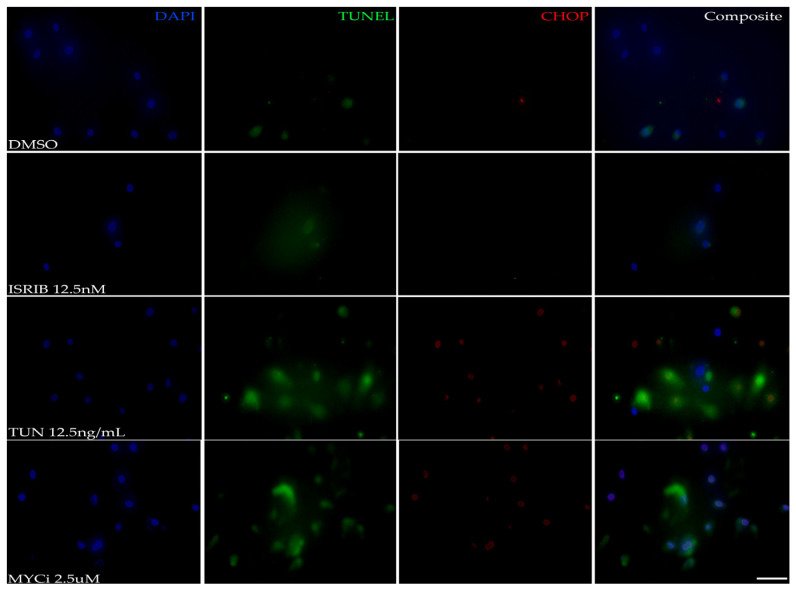
Pro-apoptotic effects of MYC inhibition in vitro. Representative photomicrographs of HMGECs incubated for eight hours in DMSO, ISRIB, tunicamycin (TUN), or MYCi361 (MYCi) (*n* = 3 chambers/treatment) subject to a TUNEL assay and CHOP immunolabeling. TUNEL staining (Alexa Fluor 488, green), indicating apoptotic cells, and CHOP expression (Alexa Fluor 555, red) were similarly increased in MYC-inhibited (MYCi) and ISR-induced (TUN) cells. DAPI (blue). Scale bar: 10 µm.

A TUNEL assay was performed to visualize fragmented DNA, which is a hallmark of apoptosis, with co-immunolabeling for CHOP. The fluorescence intensity of both the TUNEL assay and CHOP expression increased in the tunicamycin- and MYCi-treated cells in comparison to the DMSO- and ISRIB-treated cells (Figure 3), demonstrating increased apoptotic death in cells subject to stress stimuli and MYC inhibition.

### 3.2. MYC Overexpression Suppresses CHOP Expression in HMGECs

**Figure 4:**
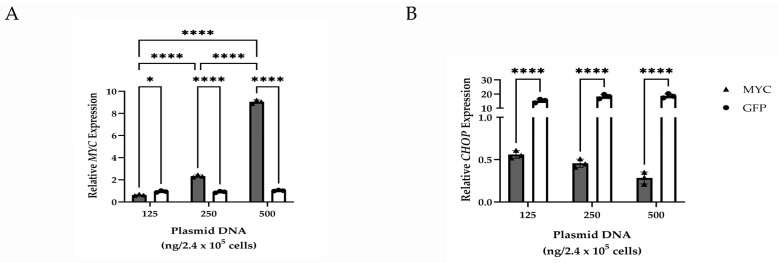
Gene expression changes resulting from MYC overexpression in vitro. Relative transcript expression of *MYC* (**A**) and *CHOP* (**B**) in HMGECs following a five-day transfection with increasing amounts of MYC or GFP plasmid DNA (*n* = 3 wells/concentration). * *p* < 0.05, **** *p* ≤ 0.0001.

To evaluate the effects of MYC overexpression on *CHOP* expression, qPCR was performed on HMGECs following a five-day transfection with a plasmid encoding the *MYC* gene. Transfection efficiency in response to variable concentrations of plasmid DNA indicated a significant (*p* ≤ 0.0001) dose-dependent increase in *MYC* expression. *MYC*-overexpressing HMGECs also exhibited significant downregulation (*p* ≤ 0.0001) of *CHOP* relative to *GFP*-transfected cells (Figure 4). Collectively, these data demonstrate an inverse relationship between *MYC* and *CHOP* expression.

### 3.3. CHOP Protein Expression and Transcript Expression Are Upregulated in MYC-Inhibited HMGECs

**Figure 5:**
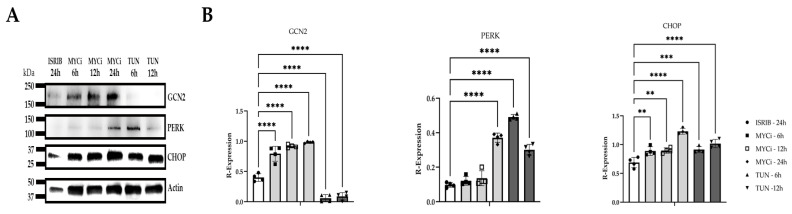
ISR-modulating effects of MYC inhibition in vitro. Relative GCN2, PERK, and CHOP expression in HMGECs following six- to twenty-four-hour incubation with ISRIB, MYCi361 (MYCi), or tunicamycin (TUN) (*n* = 4 wells/treatment/time point); (**A**). Quantification of GCN2, PERK, and CHOP protein expression normalized to actin (**B**). ** *p* < 0.01, *** *p* < 0.001, **** *p* ≤ 0.0001.

Western blotting was used to evaluate protein expression of components of the ISR including two of the implicated kinases in MYC-overexpressing tumors (GCN2, PERK) and CHOP. Densitometry demonstrated that MYC inhibition resulted in ISR induction through GCN2 activation, with significant time-dependent upregulation of GCN2 (*p* ≤ 0.0001) and CHOP (6 h: *p* = 0.0022; 12 h: *p* = 0.0014; 24 h: *p* ≤ 0.0001) following MYC inhibition (Figure 5). While PERK expression was significantly upregulated (*p* ≤ 0.0001) at the latest assessed time point of MYC inhibition (24 h), expression tended to be lower at earlier time points. Tunicamycin-treated cells exhibited cytotoxicity beginning at 12 h, with extensive cell death observed by 24 h. Protein lysates isolated from the 24 h time point were excluded from subsequent immunoblotting. Tunicamycin treatment resulted in significant upregulation of PERK (*p* ≤ 0.0001) and CHOP at the 6 h (*p* = 0.0007) and 12 h (*p* ≤ 0.0001) time points, respectively.

**Figure 6:**
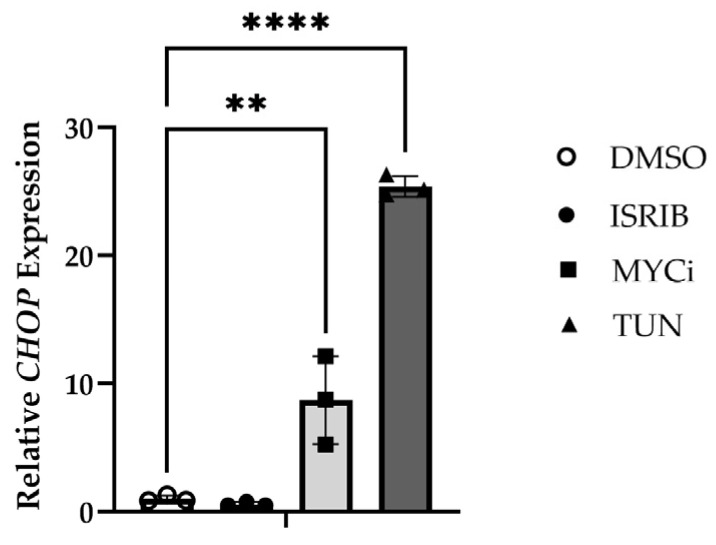
*CHOP*-inducing effects of MYC inhibition in vitro. Relative *CHOP* expression in HMGECs following a six-hour incubation with ISRIB, tunicamycin (TUN), MYCi361 (MYCi), or DMSO vehicle control (*n* = 3 wells/treatment). ** *p* < 0.01, **** *p* ≤ 0.0001.

The effects of MYC inhibition and ISR modulation on *CHOP* expression were evaluated using qPCR. HMGECs incubated with both tunicamycin (*p* ≤ 0.0001) and MYCi361 (*p* = 0.0018) exhibited significantly upregulated *CHOP* expression relative to the DMSO control and ISR-inhibition (Figure 6), confirming the relationship demonstrated at the protein level.

### 3.4. MYC Induction In Vivo Suppresses the ISR, Reduces Apoptosis, and Promotes Lipogenesis

**Figure 7:**
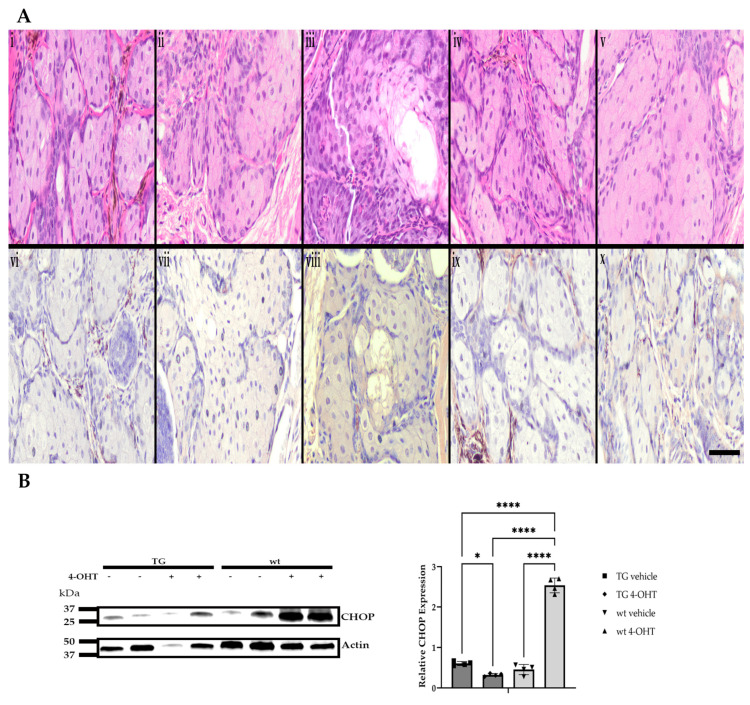
Morphologic features and CHOP expression in response to induced MYC expression in vivo. Representative photomicrographs of H&E-stained and CHOP-immunolabeled (DAB; brown) FFPE sections of murine Meibomian glands following a five-day induction with 4-hydroxytamoxifen (4-OHT) or vehicle control (*n* = 6 mice/group). Naïve wt (**i,vi**), vehicle-treated wt (**ii,vii**), 4-OHT-induced wt (**iii,viii**), vehicle-treated TG (**iv,ix**), and 4-OHT-induced TG eyelids (**v,x**). Scale bar: 25 µm (**A**). Relative protein expression of CHOP was increased in 4-OHT-treated wt mice when compared to all other groups (*n* = 4 mice/group). * *p* < 0.05, **** *p* ≤ 0.0001 (**B**).

Eyelids and periocular skin of the right eyes from both K14MycER TG mice and wt littermates were treated topically with 4-OHT for five consecutive days, while the contralateral eye served as vehicle control. FFPE eyelid tissue was then evaluated with routine H&E staining and immunohistochemistry for CHOP. Morphologically, 4-OHT-induced, *MYC*-overexpressing TG mice exhibited significantly greater mean cross-sectional areas of meibocytes (262.7 ± 88.9 µm^2^), with expanded cytoplasmic volume and demonstrable cholesterol clefts relative to 4-OHT-treated wt mice (206.1 ± 72.5 µm^2^), and vehicle-treated wt (239.4 ± 94.3 µm^2^) and TG (231.5 ± 86.1 µm^2^) mice (Figure 7A and Figure S1), suggesting a more well-differentiated phenotype in MYC-overexpressing meibocytes. CHOP expression in 4-OHT-induced TG mice was significantly lower than that in vehicle-treated TG mice (*p* = 0.0214). CHOP expression in 4-OHT-treated wt mice was significantly upregulated (*p* ≤ 0.0001) compared to all other groups (Figure 7A,B). These in vivo data further support the relationship between MYC overexpression and CHOP suppression observed in cell culture.

**Figure 8:**
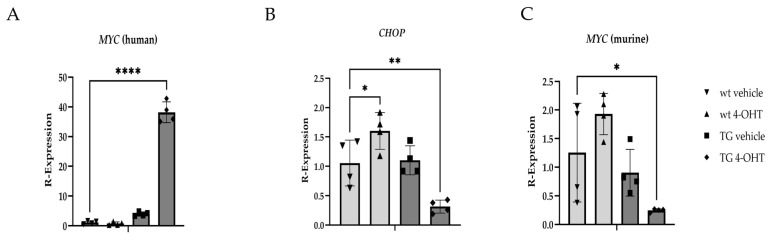
Gene expression changes resulting from induced *MYC* overexpression in vivo. Relative transcript expression of (**A**) human *MYC*; (**B**) *CHOP*; and (**C**) murine *MYC* in wildtype (wt) and K14MycER transgenic (TG) mice (*n* = 4 mice/group) treated with vehicle or 4-hydroxytamoxifen (4-OHT). * *p* < 0.05, ** *p* < 0.01, **** *p* ≤ 0.0001.

Significant differences in *MYC* expression were confirmed between wt and K14MycER TG mice treated with the vehicle and 4-OHT (Figure 8A), respectively. Transcriptional expression of *CHOP* was significantly reduced (*p* = 0.0082) in 4-OHT-induced K14MycER eyelids and significantly increased (*p* = 0.0461) in 4-OHT-treated wt tissue relative to vehicle-treated wt animals (Figure 8B), further establishing the inverse relationship between *MYC* and *CHOP*. To assess for potential effects of induction of the human *c-MYC* cDNA on native *MYC* expression, qPCR using the murine-specific probe was also performed, revealing a significant decrease (*p* = 0.0408) in *MYC* expression in 4-OHT-treated TG mice relative to vehicle-treated wt littermates (Figure 8C).

**Figure 9:**
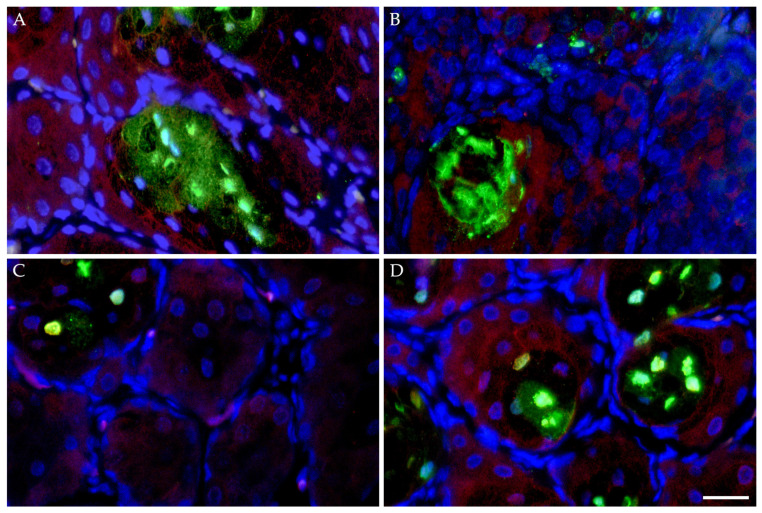
Apoptotic effects of MYC modulation in vivo. FFPE sections of murine Meibomian glands following five-day induction with 4-hydroxytamoxifen (4-OHT) with or without subsequent topical MYC inhibition (*n* = 4 mice/treatment) for three days evaluated by a TUNEL assay and CHOP immunolabeling. Wildtype mice subject to 4-OHT treatment demonstrated regionally extensive foci of apoptotic death (TUNEL, Alexa Fluor 488; green) and upregulated CHOP expression (Alexa Fluor 555; red) (**A**), both of which were mildly attenuated by subsequent MYCi361 treatment (**B**). K14MycER TG mice subject to 4-OHT induction exhibited low rates of apoptosis and downregulated CHOP expression (**C**), both exacerbated by subsequent MYCi361 treatment (**D**). DAPI (blue). Scale bar: 25 µm.

Similar to the expression patterns of the non-neoplastic HMGECs in culture, FFPE sections of K14MycER TG murine Meibomian glands subject to 4-OHT induction exhibited downregulated CHOP expression and minimal apoptotic death when evaluated by a TUNEL assay and immunolabeling (Figure 9). Both CHOP expression and apoptosis increased in response to subsequent topical treatment with MYCi361. Conversely, wt littermates subject to 4-OHT induction and topical MYC inhibition exhibited similar rates of apoptosis and intensity of CHOP expression. Collectively, these data further suggest the potent MYC-modulating capacity of both CHOP expression and subsequent apoptotic death.

FFPE sections of human ocular adnexal sebaceous carcinoma exhibited upregulated CHOP expression and brisk rates of apoptotic death in tumor foci expressing low levels of MYC, while those foci with both more robust MYC staining intensity and distribution demonstrated low CHOP expression and attenuated apoptosis (Supplementary Figure S2).

To investigate the interaction between MYC modulation and lipid production in meibocytes, HMGECs were subject to immunofluorescence to colocalize fatty acid synthase and MYC expression. Following six hours of incubation with a potent fatty acid synthase inhibitor (C75), both fatty acid synthase expression and MYC expression were suppressed in a dose-dependent manner (Supplementary Figure S3A), with cells treated in the 10 and 100 µM concentrations exhibiting membrane blebbing and nuclear condensation—key cytologic features of apoptosis. Oil-Red-O staining of frozen sections of murine eyelids subject to topical 4-OHT induction demonstrated a reduction in lipid content in wt mice, with a redistribution of lipid droplets to the more distal aspects of the acini and a substantial increase in intensity and distribution of lipid droplets in K14MycER TG mice (Supplementary Figure S3B). Morphologically, the cytoplasm of *MYC*-overexpressing TG mice was more brightly eosinophilic and vacuolated, with multifocally demonstrated clear (empty), elongated acicular spaces, consistent with cholesterol clefts (Supplementary Figure S3C). Quantitative PCR of homogenized murine tarsal plates also exhibited a significant increase (*p* = 0.0038) in the expression of *FASN* in 4-OHT-induced TG mice relative to wt littermates treated with the vehicle (Supplementary Figure S3D).

## 4. Discussion

The ISR has become a cellular pathway of interest in cancer biology due to its roles in tumor initiation, promotion, and progression and, in other contexts, its anti-tumor capabilities. Due to the complexity of the ISR pathway, cells of various lineages and differentiation differ in their response to activation and exhibit variable responses in both type and magnitude. Here, we investigated the effects of MYC expression on the ISR, specifically in the epithelial cells of the Meibomian gland.

MYC overexpression is common in many malignancies and has been reported specifically in sebaceous cell carcinomas arising from the Meibomian gland [4]. This raised the question of how MYC may interact with the ISR and how changes in MYC expression may regulate the expression of key ISR components. One critical ISR protein is CHOP, the effector responsible for inducing apoptosis when stressors exceed the capacity for cellular recovery through other feedback loops that comprise the ISR [18]. In this study, we found that MYC-inhibited HMGECs exhibit upregulated CHOP expression, similar to cells treated with tunicamycin, which is a well-documented PERK-mediated inducer of CHOP [19]. Collectively, these data suggest that MYC suppression in vitro activates the ISR, specifically leading to CHOP expression and cytotoxicity characterized by increased apoptotic cell death. Both transcriptional repression of CHOP via cofactor displacement mechanisms and potential post-transcriptional microRNA-mediated processes and chromatin modification are considered plausible mechanisms for the observed effects of MYC modulation; however, further work in this area is necessary [33]. Based on the subsequent immunoblotting of these MYC-inhibited HMGECs, CHOP expression appears to be regulated by GCN2 kinase signaling. Both CHOP and GCN2 protein expression increased in a time-dependent manner in response to pharmacologic MYC inhibition. Conversely, both *MYC*-overexpressing HMGECs and 4-OHT-induced *MYC*-overexpressing murine Meibomian glands exhibited attenuated CHOP expression and increased cytoplasmic cross-sectional areas, further suggesting a role for MYC in regulating ISR activation in this tissue.

The ability to evade apoptotic mechanisms is exceptionally advantageous during tumorigenesis [34]. Evading apoptosis allows cancer cells to continue to proliferate unchecked by normal cellular mechanisms. Esophageal neoplasms exhibiting CHOP or PERK silencing demonstrated significantly reduced hypoxia-induced apoptosis [35]. Further, chemotherapy resistance has been correlated with apoptosis evasion mechanisms [36].

While no sex-related differences in meibocyte morphology or protein or transcript expression were observed in the current study, sex-related changes in gene expression in the murine Meibomian gland have been documented, with eIF2γ expression being significantly increased in male mice relative to females (ratio: 270.3, *p* = 0.0001) [37]. Additional studies investigating the role of androgens in sex-related differences in gene expression have demonstrated that sex hormones, structurally related to cholesterol through a common central sterol nucleus, regulate the expression of hundreds to thousands of meibocyte genes [38,39]. The influence of aromatase expression on murine Meibomian gland morphology has been investigated, but a specific role for selective estrogen receptor modulators (SERM; i.e., 4-OHT) remains to be fully characterized [40]. Critically, the effects observed in the current study cannot be attributed to SERMs alone, as differential effects were noted in response to inducible MYC expression. While the use of topical 4-OHT, permitting both spatial and temporal regulation of *MYC* expression in our in vivo mouse model, was not specifically assessed for its potential to induce cell stress independent of MYC modulation, the ISR-inductive and robust apoptotic effects observed in wt mice were not present to the same extent in the TG mice. These findings suggest that the topical application of 4-hydroxytamoxifen alone does not activate the ISR or apoptotic pathways equivalently in the context of *MYC* overexpression relative to a more constitutive baseline. Interestingly, in human breast cancer cells, tamoxifen has been shown to be protective against ER stresses induced by nutrient deprivation through the enhancement of a specialized form of autophagy to support neoplastic cell survival in the face of nutrient depletion in the tumor microenvironment [41].

Further complicating the analyses of the relationships between cell stress response and cell death are the complex and broadly reaching impacts of MYC on apoptotic pathways, with well-documented pro- or anti-apoptotic depending on cell type and signaling stimulus. In p53-independent mechanisms, MYC is capable of both suppressing the Bcl-2 family of proteins to limit apoptosis while also upregulating the expression of BIM and Bax to promote apoptosis [42]. Additionally, MYC can promote p53 expression by activation of ARF to effectively limit tumor development by accelerating apoptotic cell death [43]. To better interrogate the specific role for MYC in evading apoptosis in the Meibomian gland, we then investigated the intersection of MYC and lipogenesis due to the meibum-synthesizing functions of these cells [44]. MYC plays an integral role in regulating the conversion of glucose to acetyl-CoA and then to palmitate, and the maintenance of acetyl-CoA in support of membrane synthesis is essential for proliferating cells, especially in the face of ER stress [45,46]. In both our in vitro experiments with fatty acid synthase-inhibited HMGECs and in our in vivo studies assessing intracellular lipid accumulation and *FASN* expression in murine Meibomian glands, an interplay between lipogenesis and MYC expression was demonstrated.

Dysregulated lipid metabolism is widely documented in benign and malignant neoplasms, and MYC, in collaboration with sterol regulated element-binding protein (SREBP), has been shown to regulate lipogenesis in the promotion of tumorigenesis [47]. The effects of peroxisome proliferator-activated receptor gamma (PPARγ) in lipid metabolism, adipocyte differentiation, and apoptosis have been shown to be augmented by MYC inhibition, resulting in more well-differentiated phenotypes in both prostatic carcinoma and other neoplasms [48,49,50]. Pharmacologic inhibition of fatty acid synthase has been shown to trigger apoptosis during the S phase in human breast cancer cells, with C75-induced disruptions in phospholipid synthesis [51]. Gouw et al. further demonstrated that inhibiting fatty acid synthesis resulted in both tumorigenesis prohibition and xenograft regression in various MYC-induced tumor models [47]. The role of MYC in the regulation of glucose and glutamine metabolism is well-documented, but MYC has also been identified as a primary driver of carbon incorporation from these compounds into de novo synthesized fatty acids [47]. For this reason, high-MYC-expressing neoplastic cells, including three recently established primary sebaceous carcinoma cell lines, have demonstrated sensitivity to glutamine analogs following the selective suppression of the metabolic reactions that utilize glutamine [5,52]. Several studies have also identified links between ER stress and lipogenesis, with many of the same proteases responsible for ISR activation also exhibiting SREBP processing activity, and one group reported that in their screen for small molecule activators of the ISR, two candidates also demonstrated SREBP activating potential [53,54,55,56]. In an investigation of the interaction between lipid metabolism and protein-induced stress, Garcia et al. reported that knockout of a hydroxysteroid dehydrogenase, a critical catalyst in steroid hormone metabolism, resulted in both reduced lipid stores and inhibition of the UPR [57]. Little et al. also demonstrated robust PERK-mediated eIF2α activation in various mammalian tumor cell lines in response to FASN inhibition [58]. Collectively, these data and additional studies support the premise that stress-induced alterations in lipid metabolism and composition affect the membrane dynamics and survival of cancer cells [59,60,61].

Few studies have investigated ISR activation or MYC expression in the specific context of the Meibomian gland; however, downregulation of *MYC* has been implicated as a potential contributing factor to the development of Meibomian gland dysfunction (MGD), and Liu et al. suggested that genes involved in keratinocyte differentiation are associated with MGD when compared to normal HMGECs [62,63,64,65]. Further, osmotic stress of the ocular surface, with subsequent induction of the ISR, has been proposed as a decisive component in the development of MGD, further supporting our suggestion of a potentially novel axis between MYC expression, ISR activity, and lipogenesis in the Meibomian gland [66].

There are several limitations to the current study. Primarily, the effects of high-MYC expression by neoplastic meibocytes on the ISR were not evaluated to determine whether these trends are conserved in the context of malignancy; however, work to evaluate the ISR in three primary human ocular adnexal sebaceous gland carcinoma cell lines to elucidate how the MYC-ISR axis affects meibocyte proliferation, tumorigenesis, and apoptosis is ongoing [5]. With respect to our in vivo studies, 4-OHT-induced *MYC* expression alone was not sufficient to generate a tumor phenotype, and the existence of animal models capable of recapitulating the features observed in human SebCA patients is lacking [5,67]. Additionally, while confocal microscopy was not available in the current study, this technique may represent a more sensitive approach to determining morphologic changes, such as three-dimensional volume, in the murine Meibomian gland [68]. While TUNEL assays were pursued to evaluate changes in apoptosis related to MYC modulation, assessments of MYC-mediated autophagy in our in vitro and in vivo models have yet to be performed. Finally, the specific ISR-activating kinases responsible for phosphorylation of eIF2α in the context of MYC modulation in vivo and interrogating whether ISR effectors may serve as biomarkers for MYC activity in SebCA requires further investigation and represents an exciting field for future study.

## 5. Conclusions

Here, we aimed to characterize the role of MYC in the modulation of the ISR within Meibomian gland epithelial cells using both pharmacologic and genetic approaches. MYC inhibition in HMGECs led to an upregulation of CHOP at both the protein and transcript level, along with the hallmarks of apoptotic cell death including induced LDH release and DNA fragmentation. In the K14MycER mouse model, we also demonstrated attenuated CHOP expression and increased intracellular lipid accumulation and *FASN* expression in response to MYC overexpression. This may indicate that MYC expression plays a crucial role in modulating the ISR, diverting signal propagation away from apoptotic pathways and stimulating fatty acid synthesis, mechanisms that may be critical in the tumorigenesis of the Meibomian gland.

## Data Availability

The original contributions presented in this study are included in the article Supplementary Materials. Further inquiries can be directed to the corresponding author.

## Supplementary Materials

The following supporting information can be downloaded at https://www.mdpi.com/article/10.3390/cells14100709/s1, Figure S1: Morphologic and morphometric assessments of the murine Meibomian gland in response to MYC modulation; Figure S2: Apoptotic effects of MYC modulation in sebaceous carcinoma; Figure S3: Interplay between MYC modulation and lipogenesis in vitro and in vivo.
