# Supplementary material for: A Potential Role for c-MYC in the Regulation of Meibocyte Cell Stress
Source: Cells. 2025 May 14;14(10):709. doi: 10.3390/cells14100709 (PMC12109776; doi:10.3390/cells14100709)
Supplement: Supplementary file 1 [file cells-14-00709-s001.zip › Boyack et al Supplementary Matierals.pdf]

cells MDPI

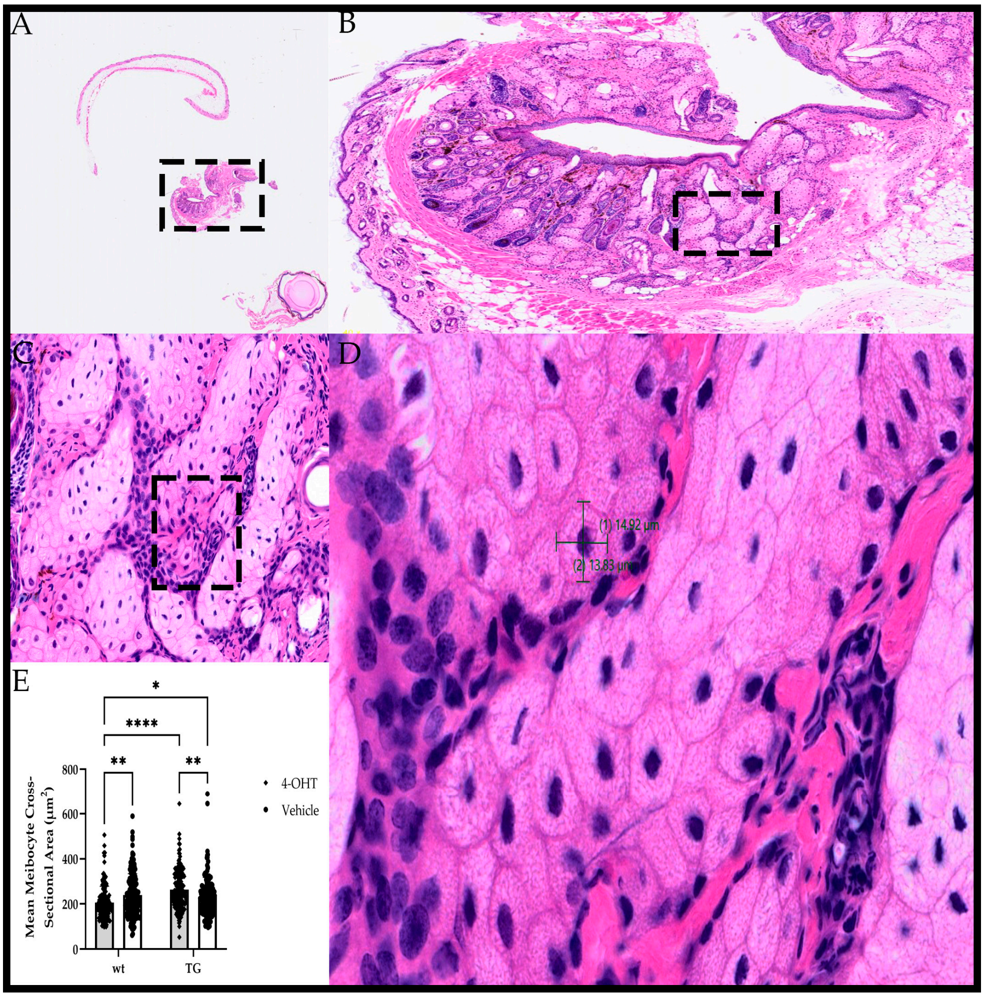


**Supplementary Figure 1. Morphologic and morphometric assessments of the murine Meibomian gland in response to MYC modulation.** Representative whole slide images (WSIs) of H&E-stained FFPE sections of murine tissues. Representative 0.1x magnified slide image demonstrating dorsal skin (top), eyelid with periocular skin (boxed region), and globe (bottom (**a**). Representative 1.6x magnified slide image of the eyelid demonstrating skin and conjunctival surfaces with Meibomian gland acini (boxed region). (**b**) Representative 8x magnified slide image demonstrating Meibomian glands with peripheral basal cells and more central meibocytes with sebaceous differentiation. (**c**) Representative 40x magnified slide image demonstrating a field containing polygonal to polyhedral meibocytes. One meibocyte is labeled with representative orthogonal measurements (**d**). Mean cross sectional area of meibocytes ($n = 10$ cells/acini; 4 acini/mouse) in wildtype (wt) and K14MycER transgenic (TG) mice ($n = 4$/group) treated with vehicle control or 4-hydroxytamoxifen (4-OHT). *$p < 0.05$, **$p < 0.01$, ****$p \leq 0.0001$.

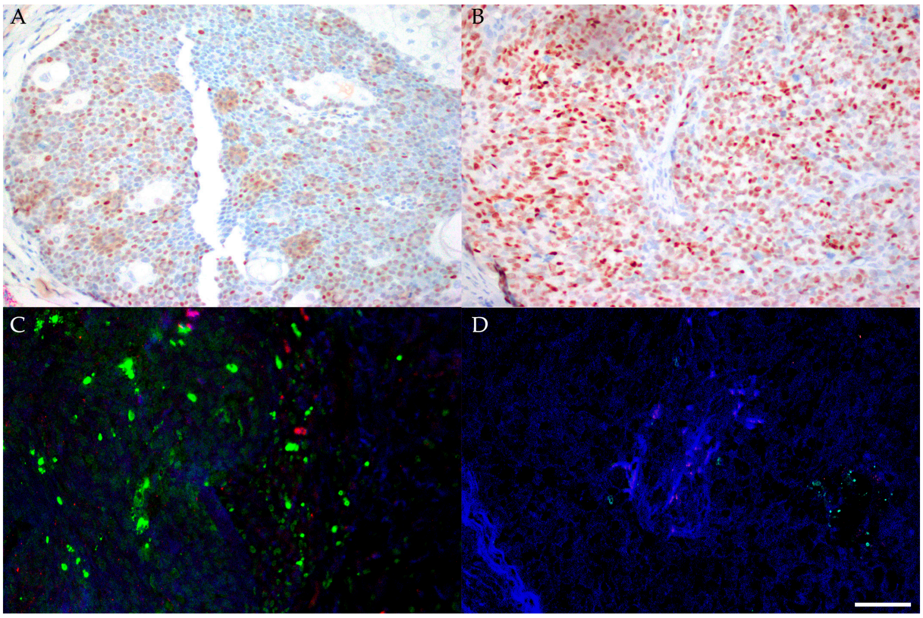


**Supplementary Figure 2. Apoptotic effects of MYC modulation in sebaceous carcinoma.** FFPE sections of human primary ocular adnexal sebaceous carcinoma exhibiting either low MYC (DAB; brown) expression as determined by H-score(**a**) or high MYC expression (**b**) (*n* = 3 tumors/MYC expression stratification). Low MYC-expressing tumors exhibited upregulated CHOP expression (Alexa Fluor 555; red) and brisk rates of apoptotic death (TUNEL, Alexa Fluor 488; green) (**c**), while high MYC-expressing tumors demonstrated suppressed CHOP expression and attenuated apoptosis (**d**). DAPI (blue). Scale bar: 50μm.

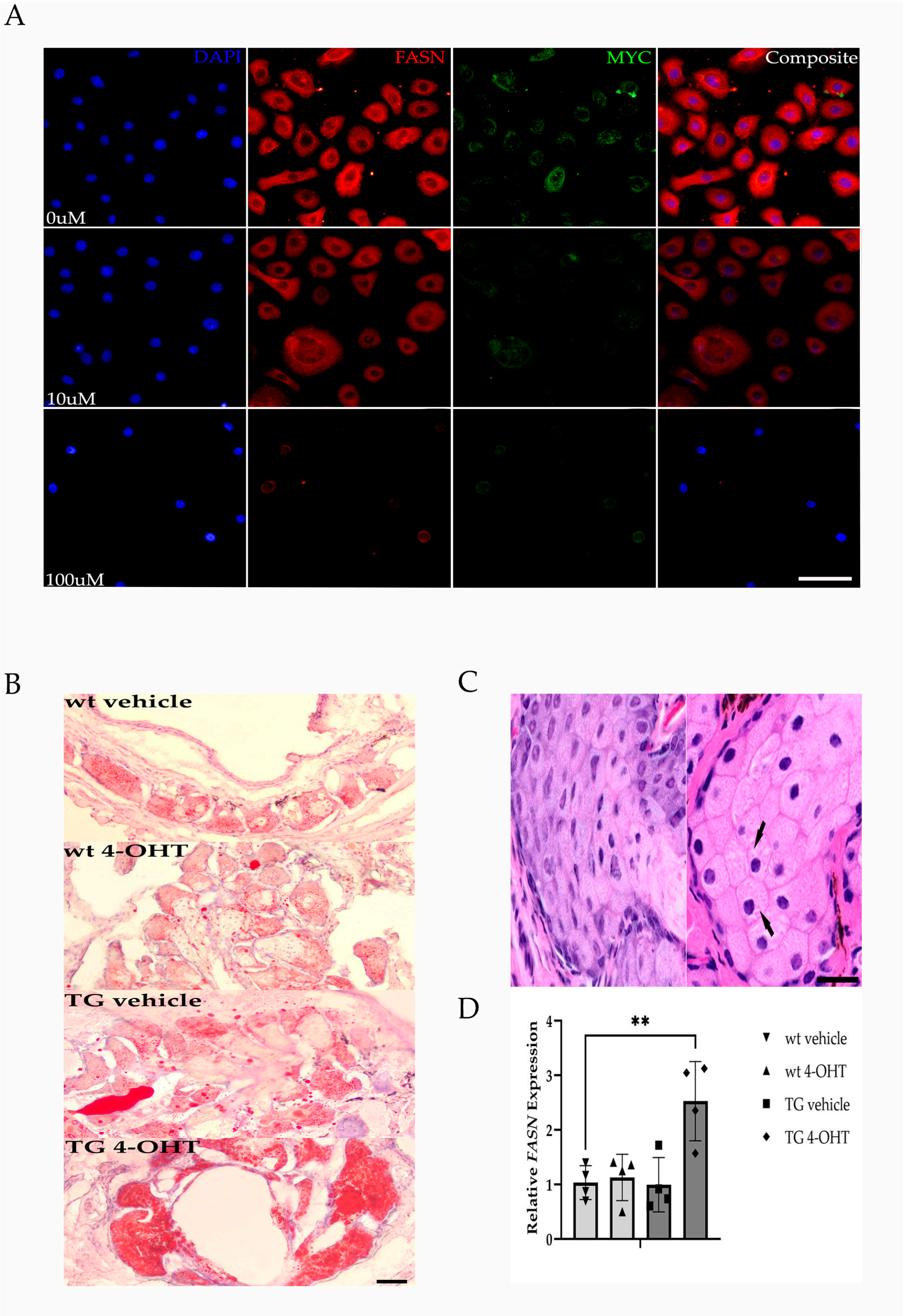


**Supplementary Figure 3. Interplay between MYC modulation and lipogenesis *in vitro* and *in vivo*.** Representative photomicrographs of HMGECs incubated for six hours in C75 at concentrations ranging from 0-100µM (*n* = 3 chambers/treatment) subject to immunolabeling for fatty acid synthase (FASN) expression (Alexa Fluor 555, red) and MYC (Alexa Fluor 488, green) demonstrating dose-dependent downregulation of both proteins. DAPI (blue). Scale bar: 25µm (**a**). Representative photomicrographs of Oil-Red-O-stained frozen sections of wildtype (wt) and K14MycER transgenic (TG) eyelid following five days of treatment with vehicle control or 4-hydroxytamoxifen (4-OHT) highlighting cytoplasmic lipid (red). Scale bar: 50µm (**b**). Representative slide images of H&E-stained FFPE sections of wildtype (left) and transgenic (right) Meibomian glands following five days of treatment with 4-OHT, highlighting the relative abundance of cytoplasmic lipids and presence of

cholesterol clefts (arrows) in 4-OHT induced TG mice. Scale bar: 25µm (**c**). Relative transcript expression of *FASN* in wt and TG mice ($n$ = 4 mice/group) treated with vehicle or 4-OHT. **$p < 0.01$ (**d**).
