# Supplementary figures and images for: A Potential Role for c-MYC in the Regulation of Meibocyte Cell Stress
Source: Cells. 2025 May 14;14(10):709. doi: 10.3390/cells14100709 (PMC12109776; doi:10.3390/cells14100709)

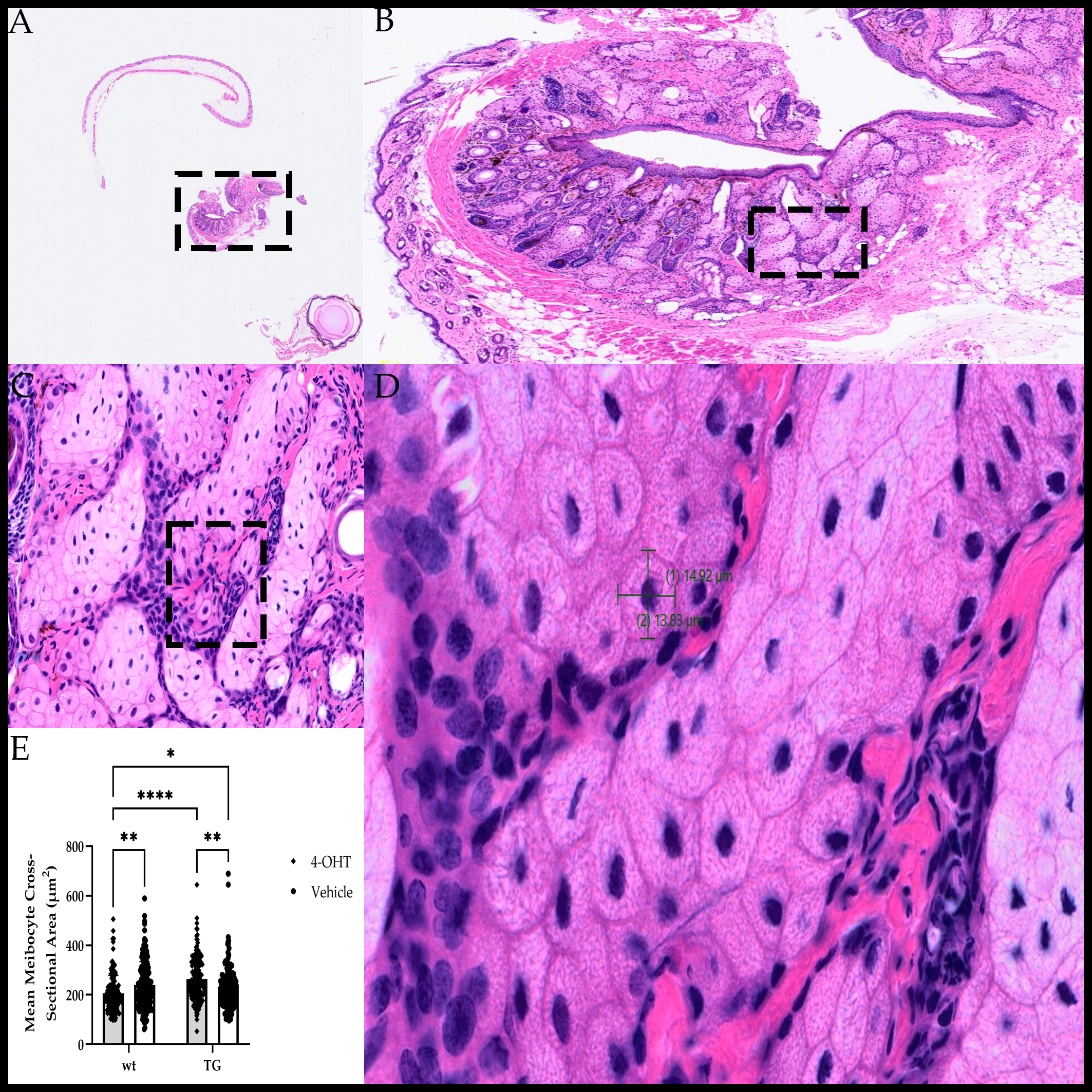

Supplement: Supplementary file 1 [file cells-14-00709-s001.zip › Boyack et al Fig S1.tif]

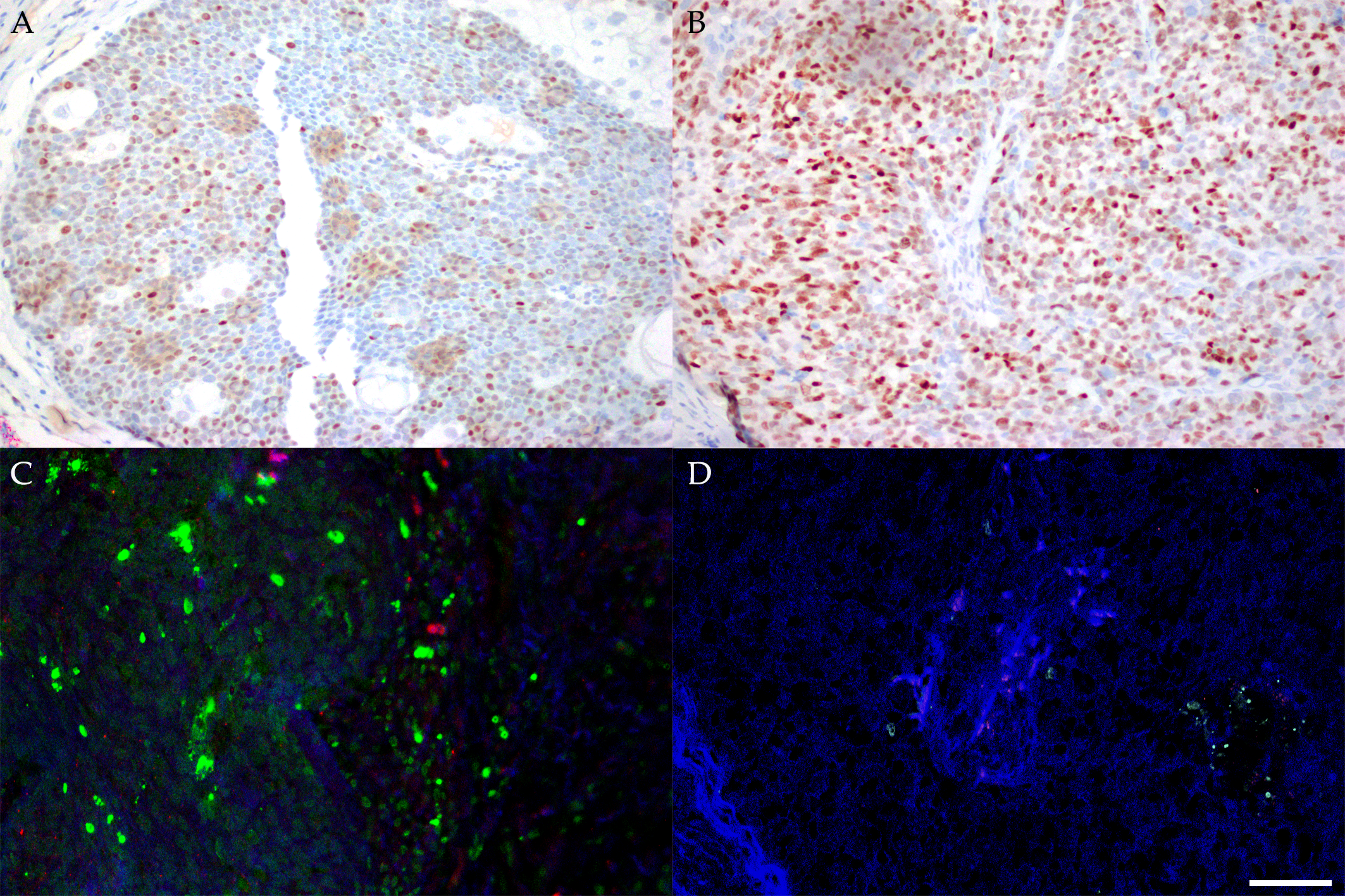

Supplement: Supplementary file 1 [file cells-14-00709-s001.zip › Boyack et al Fig S2.tif]

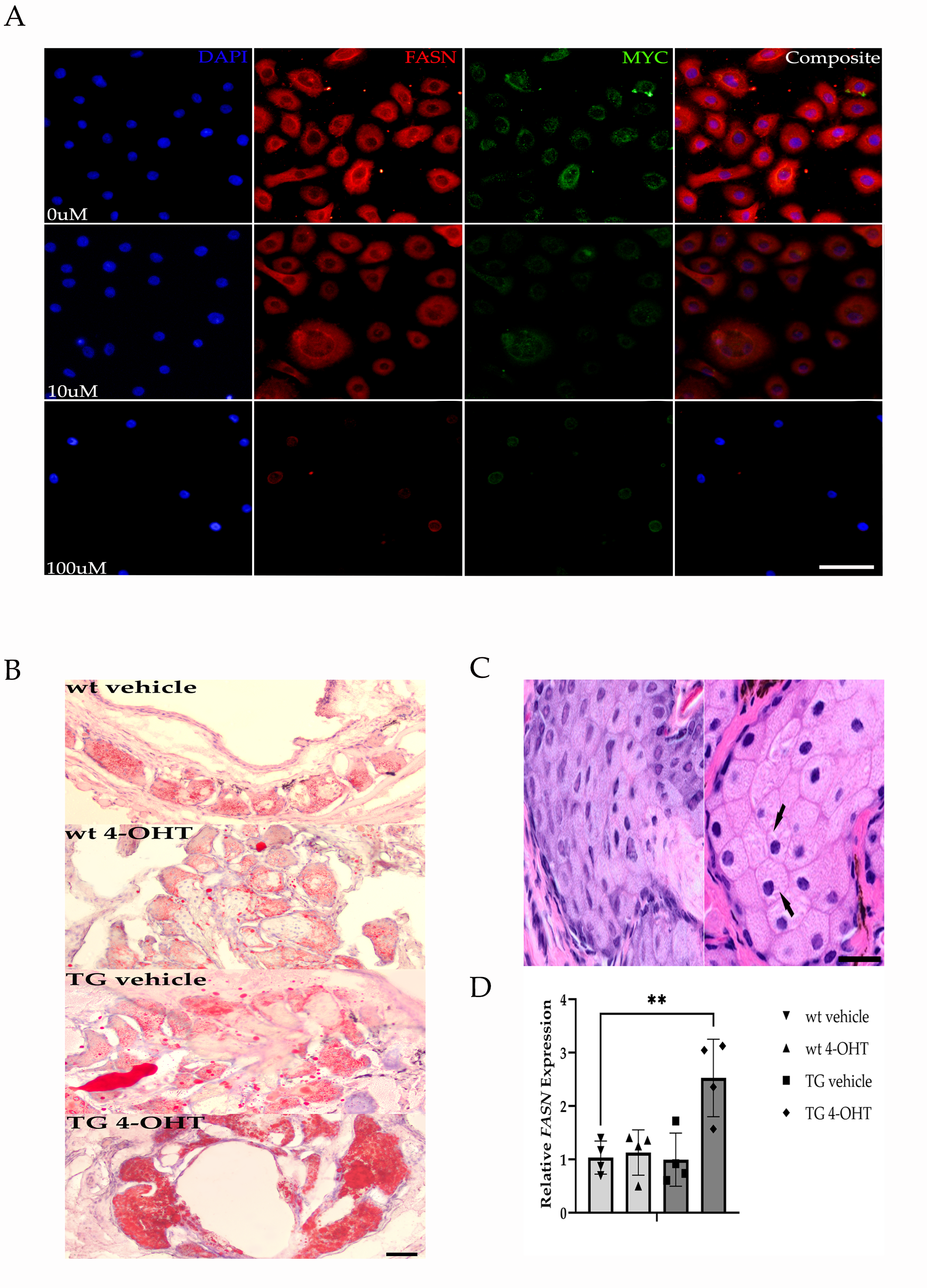

Supplement: Supplementary file 1 [file cells-14-00709-s001.zip › Boyack et al Fig S3.tif]
